# Activation of locus coeruleus noradrenergic neurons rapidly drives homeostatic sleep pressure

**DOI:** 10.1126/sciadv.adq0651

**Published:** 2025-01-17

**Authors:** Daniel Silverman, Changwan Chen, Shuang Chang, Lillie Bui, Yufan Zhang, Rishi Raghavan, Anna Jiang, April Le, Dana Darmohray, Jiao Sima, Xinlu Ding, Bing Li, Chenyan Ma, Yang Dan

**Affiliations:** Department of Neuroscience, Helen Wills Neuroscience Institute, Howard Hughes Medical Institute, University of California, Berkeley, Berkeley, CA 94720, USA.

## Abstract

Homeostatic sleep regulation is essential for optimizing the amount and timing of sleep for its revitalizing function, but the mechanism underlying sleep homeostasis remains poorly understood. Here, we show that optogenetic activation of locus coeruleus (LC) noradrenergic neurons immediately increased sleep propensity following a transient wakefulness, contrasting with many other arousal-promoting neurons whose activation induces sustained wakefulness. Fiber photometry showed that repeated optogenetic or sensory stimulation caused a rapid reduction of calcium activity in LC neurons and steep declines in noradrenaline/norepinephrine (NE) release in both the LC and medial prefrontal cortex (mPFC). Knockdown of α_2_A adrenergic receptors in LC neurons mitigated the decline of NE release induced by repetitive stimulation and extended wakefulness, demonstrating an important role of α_2_A receptor–mediated auto-suppression of NE release. Together, these results suggest that functional fatigue of LC noradrenergic neurons, which reduces their wake-promoting capacity, contributes to sleep pressure.

## Teaser

Repeated activation of locus coeruleus neurons increases sleep drive partly due to their reduced ability to release noradrenaline.

## INTRODUCTION

Sleep, an innate behavior essential for our survival (*1*, *2*), is known to be regulated homeostatically: The propensity to sleep and difficulty of awakening (sleep pressure) increase with the amount of previous wakefulness (*3*). Several mechanisms of sleep pressure have been proposed (*4*, *5*), including wake-dependent elevation of extracellular adenosine, which can inhibit wake-promoting neurons (*6*–*8*); synaptic strengthening, which can enhance slow-wave synchrony during nonrapid eye movement (NREM) sleep (*9*); and phosphorylation of the proteome and alteration of the transcriptome in the brain (*10*–*14*). However, the extent to which each of these mechanisms contributes to homeostatic regulation of natural sleep-wake cycles remains unclear.

Here, we explore the possibility that activity-induced functional fatigue of wake-promoting neurons contributes to homeostatic sleep pressure. We focused on locus coeruleus (LC) neurons that release NE, which strongly promote wakefulness and arousal (*15*–*17*) and are generally more active during wakefulness than sleep (*18*–*20*). Using optogenetic and sensory stimulation combined with fiber photometry, we characterized evoked calcium responses and NE release from LC-NE neurons as well as their effects on sleep-wake states.

## RESULTS

### Optogenetic activation of LC-NE neurons increases sleep pressure

**Fig. 1.**
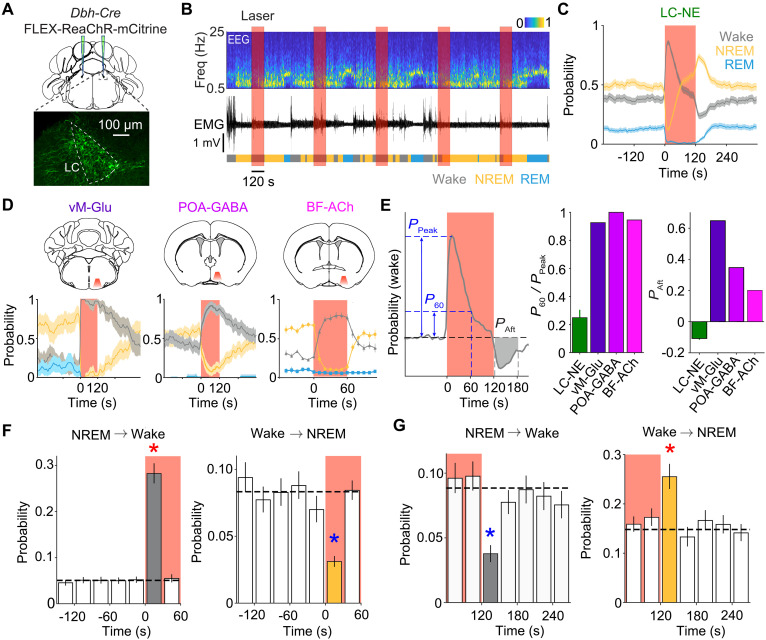
Optogenetic activation of LC-NE neurons drives rebound sleep following transient wakefulness. (**A**) Schematic for optogenetic activation of LC-NE neurons and fluorescence image showing ReaChR-mCitrine expression (intrinsic mCitrine fluorescence). (**B**) Example session showing EEG power spectrum (top), EMG (middle), and color-coded brain states (bottom). The EEG power at each frequency in each time bin (5 s/bin) was normalized by the maximum of the entire recording session; thus, the value varies between 0 and 1 (upper right scale bar). (**C**) Probability of wake, NREM, and REM states before, during, and after laser stimulation (mean and 95% confidence interval, *n* = 8 mice). Red shading, laser stimulation period. (**D**) Similar to (C) but for stimulating glutamatergic neurons in the ventral medulla (vM-Glu, 2 min), GABAergic neurons in the preoptic area (POA-GABA, 2 min), and cholinergic neurons in the basal forebrain (BF-ACh, 1 min); data reproduced from (*21*–*23*). (**E**) Comparison of wake-promoting effects of the four populations. Left: Schematic illustrating parameters for quantification. Middle: Persistence of wake effect measured by *P*_60_/*P*_Peak_ ratio. Right: Aftereffects of optogenetic activation (*P*_Aft_). (**F**) NREM→wake and wake→NREM transition probabilities before and after stimulation onset. (**G**) Similar to (F), but before and after termination of stimulation. Dashed line, mean of baseline period (−240 to 0 s). Red star, significant increase, *P* < 0.0001; blue star, significant decrease, *P* < 0.0001 (bootstrap).

To measure the effects of activating LC-NE neurons on sleep-wake brain states, we injected a Cre-inducible adeno-associated virus (AAV) expressing a red-shifted channelrhodopsin (AAV2-hSyn-FLEX-ReaChR-mCitrine) bilaterally into the LC of *Dbh-Cre* mice (Fig. 1A). During each recording session (8 a.m. to 12:30 p.m.), laser stimulation (10 mW, 532 or 589 nm, 10-ms pulses at 10 Hz or a 2-s pulse every 10 ± 5 s) was applied in 2-min episodes, repeated every 10 min, and sleep-wake states were classified based on electroencephalogram (EEG) and electromyogram (EMG) recordings (Fig. 1B and fig. S1A). We found a strong increase in wakefulness within a few seconds after the onset of each stimulation episode (Fig. 1C), consistent with the known wake-promoting effect of LC neurons (*15*–*17*). However, the increase in wakefulness was highly transient: Even within the 2-min stimulation episode, the probability of wakefulness declined to nearly the baseline level, and immediately after the stimulation, wakefulness fell below the baseline, accompanied by increases in NREM and REM sleep. Similarly, transient waking effects were observed with unilateral laser stimulation (fig. S1B) and across different temporal patterns and frequencies of stimulation known to evoke spiking reliably in brain slices (*15*) and in vivo (*16*) (fig. S1, B to F). In control mice expressing green fluorescent protein (GFP) without ReaChR, laser stimulation had no effect (fig. S1J).

Such a transient wake-promoting effect of LC neuron activation is in stark contrast to the effects of similar optogenetic stimulation of glutamatergic neurons in the ventral medulla (vM-Glu) (*21*), GABAergic neurons in the preoptic area (POA-GABA) (*22*), and cholinergic neurons in the basal forebrain (BF-ACh) (*23*), all of which were previously shown to induce wakefulness that outlasted the duration of laser stimulation (published figures reproduced in Fig. 1D). To quantify the persistence of the wake effect induced by optogenetic activation, we computed the ratio between the increase in wake probability at 60 s after laser onset (*P*_60_) and the peak increase within the laser episode (*P*_Peak_) (Fig. 1E, left). The *P*_60_/*P*_Peak_ ratio for LC-NE neurons was 0.25 ± 0.06 (mean ± SEM, *n* = 8 mice), indicating a strong decay in the wake effect at 60 s after stimulation onset. In contrast, *P*_60_/*P*_Peak_ values for vM-Glu, POA-GABA, and BF-ACh neurons were all >0.9 (Fig. 1E, middle). To quantify the aftereffect of the stimulation episode, we computed the difference between the mean wake probability 0 to 60 s after the stimulation and the baseline period before stimulation (*P*_Aft_). Whereas *P*_Aft_ values for vM-Glu, POA-GABA, and BF-ACh neurons were all > 0, indicating persistence of the wake effect beyond the stimulation episode, *P*_Aft_ for LC-NE neurons was −0.11 ± 0.01, indicative of rebound sleep after the stimulation (Fig. 1E, right).

To further quantify the effects of LC-NE neuron activation, we calculated the probabilities of NREM→wake and wake→NREM transitions before, during, and after the stimulation episode. Compared to the baseline period before stimulation, laser stimulation increased the NREM→wake transition and decreased the wake→NREM transition, but only within the first 30 s of the 2-min episode (Fig. 1F; *P* < 0.0001, bootstrap). Immediately after stimulation, the NREM→wake transition decreased and wake→NREM transition increased significantly (Fig. 1G; *P* < 0.0001), indicating increased sleep drive. In addition to the probabilities of sleep-wake states and their transitions, we also analyzed EEG δ power during NREM sleep, a common measure of homeostatic sleep pressure. After a transient decrease at the onset of stimulation, δ power quickly rebounded and rose significantly above the baseline level immediately after stimulation during both NREM sleep and wakefulness (fig. S1H and table S1; *P* < 0.01). Together, the increases in both NREM probability and EEG δ power indicate an elevation of sleep pressure after minutes of LC neuron activation.

### Optogenetic activation of LC-NE neurons leads to their functional fatigue

**Fig. 2.**
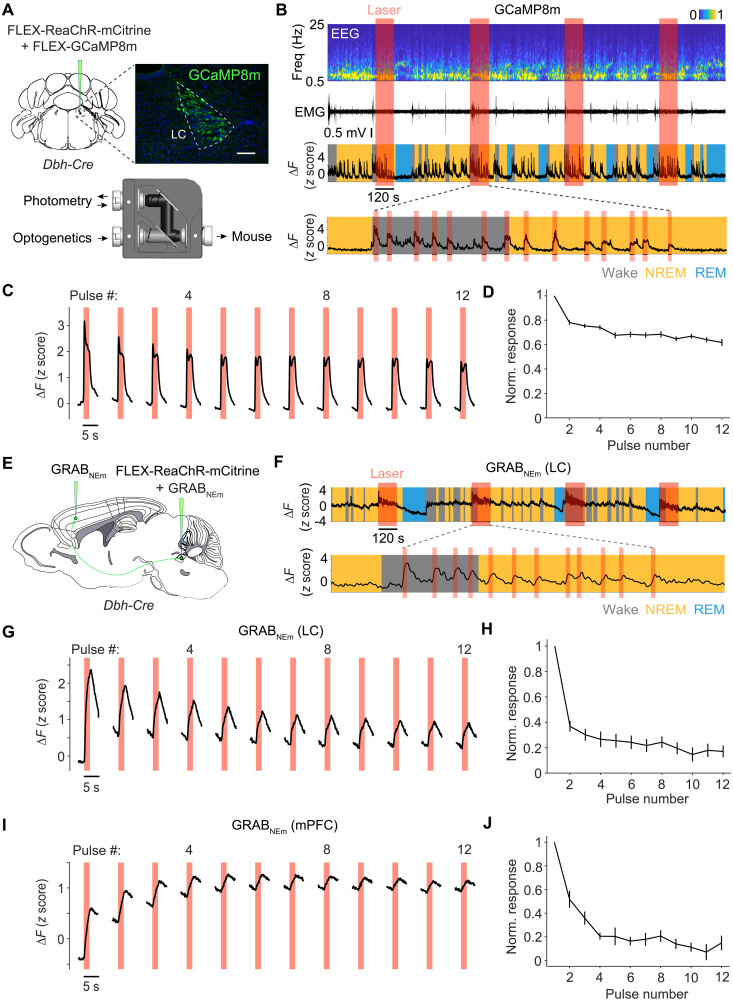
Calcium activity of LC-NE neurons and NE release evoked by optogenetic activation. (**A**) Top: Coronal diagram for virus injection into the LC of *Dbh-Cre* mice and fluorescence image showing GCaMP8m expression (immunostaining, in a mouse expressing GCaMP8m without mCitrine). Scale bar, 100 μm. Bottom: Optical pathway for simultaneous optogenetic stimulation and fiber photometry. (**B**) Example session showing calcium activity in LC-NE neurons during optogenetic stimulation. Expanded view of one stimulation episode shows calcium responses to individual 2-s pulses. (**C**) Calcium responses to individual 2-s pulses averaged across all episodes and all mice (*n* = 6 mice). (**D**) Summary of response amplitude (normalized by the maximal response in each episode, mean ± SEM). (**E**) Sagittal diagram for injection of ReaChR-mCitrine and GRAB_NEm_ into the LC and GRAB_NEm_ into the mPFC of Dbh-Cre mice. (**F**) NE release in the LC evoked by optogenetic activation. (**G** to **J**) Similar to (C) and (D), but for NE responses in the LC [(G) and (H); *n* = 7 mice] and mPFC [(I) and (J); *n* = 6 mice].

A potential mechanism for the rapid decrease in wake-promoting effect of LC-NE neurons is an attenuation of their activity caused by repeated optogenetic activation. We next measured calcium activity of LC-NE neurons in response to laser stimulation. Cre-inducible AAVs expressing jGCaMP8m [AAV9-FLEX-jGCaMP8m, (*24*)] and ReaChR [AAV2-FLEX-ReaChR-mCitrine, (*25*)] were injected into the LC of *Dbh-Cre* mice, and optogenetic stimulation and fiber photometry imaging were performed simultaneously through the same optic fiber (Fig. 2A). Coimmunostaining of jGCaMP8m and tyrosine hydroxylase (TH; an LC-NE neuron marker) showed that jGCaMP8m was expressed in 64 ± 5% of TH-positive LC-NE neurons (mean ± SEM, *n* = 4 mice). Very few jGCaMP8m-expressing neurons were TH-negative (29 of 377 across all mice), consistent with the specificity of the *Dbh-Cre* line for labeling LC-NE neurons (*26*). Without optogenetic stimulation, LC-NE neurons showed higher activity during wakefulness compared to both NREM and REM sleep (fig. S2), consistent with previous studies (*18*–*20*). Each 2-s laser pulse evoked a salient calcium response (Fig. 2B), but the response amplitude decreased consistently over successive laser pulses within each episode (Fig. 2C). For pulse 12 (near the end of each episode), the response amplitude was only 0.62 ± 0.03 (SEM) of the response to pulse 1 (Fig. 2D).

**Fig. 3.**
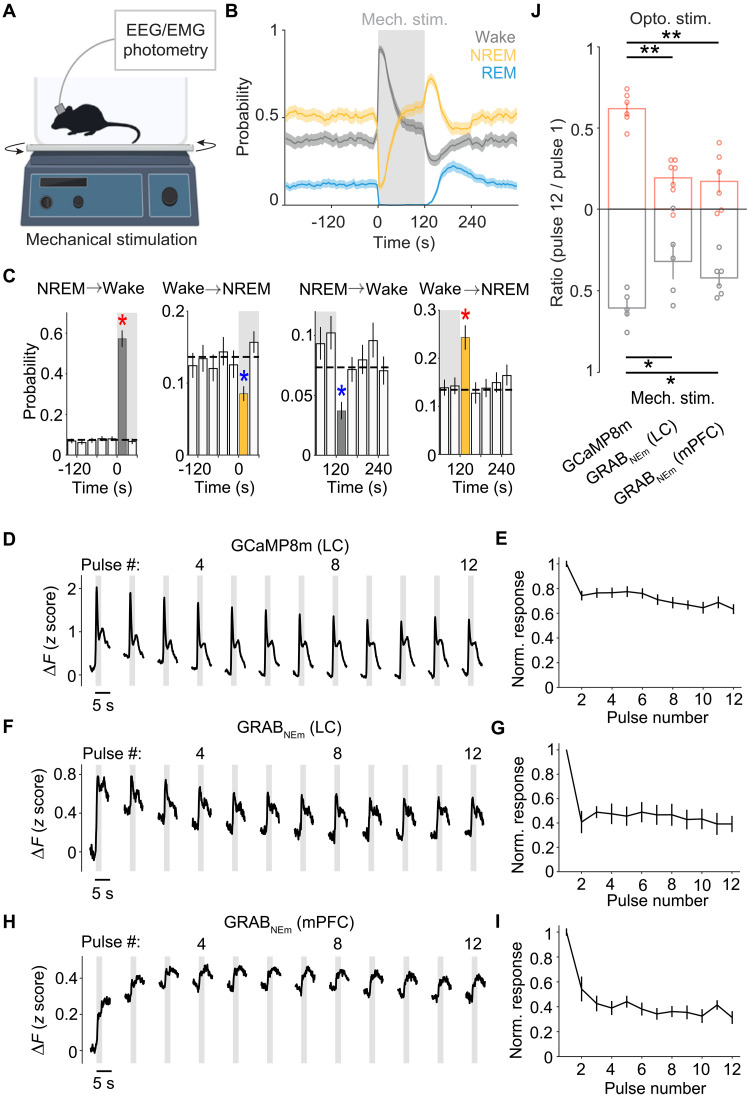
Effects of sensory stimulation on brain states, LC neuron calcium activity, and NE release. (**A**) Schematic of mechanical stimulation with an orbital shaker. (**B**) Sleep-wake probability before, during, and after 2-min mechanical stimulation (mean and 95% confidence interval, *n* = 15 mice). (**C**) NREM→wake and wake→NREM transition probabilities immediately before and after the onset (left) and termination (right) of stimulation. Red star, significant increase, *P* < 0.0001; blue star, significant decrease, *P* < 0.0001 (bootstrap). (**D** and **E**) Calcium responses of LC-NE neurons to consecutive pulses of mechanical stimulation (*n* = 5 mice), similar to Fig. 2 (C and D). (**F** to **I**) Similar to (D) and (E) but for mechanical stimulation-evoked NE release in the LC [(F) and (G); *n* = 5 mice] and mPFC [(H) and (I); *n* = 6 mice]. (**J**) Summary of functional fatigue induced by optogenetic (top) and sensory stimulation (bottom), quantified by pulse 12/pulse 1 ratio for calcium responses and NE release (mean ± SEM). *P < 0.05 **P < 0.01 (one-sided, Wilcoxon rank-sum test).

We next measured laser-evoked NE release by expressing a G protein–coupled receptor (GPCR) activation–based fluorescent NE sensor (GRAB_NEm_) (*27*) in the LC or the medial prefrontal cortex (mPFC) and ReaChR in LC-NE neurons (Fig. 2E). Each 2-s laser pulse evoked a rapid increase in NE level (Fig. 2F), but the response amplitude also declined progressively in both the LC (Fig. 2, G and H) and the mPFC (Fig. 2, I and J). The decrease in evoked NE release measured by the ratio between pulse 12 and pulse 1 (LC: 0.17 ± 0.03; mPFC: 0.15 ± 0.06; mean ± SEM) was much greater than the decrease in calcium responses (Fig. 3J), indicating a stronger decline of the functional output of these neurons. Thus, repeated LC neuron activation caused a rapid “functional fatigue”—evidenced by diminished calcium activity and NE release—which may contribute to the loss of their wake-promoting effect within each stimulation episode (Fig. 1F). The sleep rebound immediately after stimulation (Fig. 1, C and G) is likely caused by a reduction of spontaneous LC-NE activity following the 2-min optogenetic activation.

### Effects of sensory stimulation

Since optogenetic stimulation could potentially induce nonphysiological patterns of neuronal activity, we wondered whether repeated sensory stimulation could also induce LC neuron fatigue and homeostatic sleep pressure. The mouse was recorded in its home cage on top of an orbital shaker (Fig. 3A), which provided mechanical stimulation (cage shaking) in the same temporal pattern as optogenetic stimulation (2-s stimulation every 10 ± 5 s, for 2 min/episode). Such mechanical stimulation also induced a transient wakefulness followed by a sleep rebound (Fig. 3, B and C, and fig. S3H), with a time course similar to that induced by optogenetic stimulation of LC-NE neurons (Fig. 1, C, F, and G). Furthermore, calcium activity of LC-NE neurons (Fig. 3, D and E) and NE release in the LC (Fig. 3, F and G) and mPFC (Fig. 3, H and I) evoked by each 2-s mechanical stimulation decreased progressively over the 2-min episode, and the decrease in NE release was significantly stronger than the decrease in calcium responses (Fig. 3J). Thus, repeated sensory stimulation can also induce functional fatigue of LC-NE neurons as well as homeostatic sleep pressure, similar to the effects of optogenetic stimulation.

When optogenetic and sensory stimulation episodes were applied consecutively (fig. S3, A to C), the wake-promoting effect of sensory stimulation was significantly reduced by the preceding optogenetic stimulation (fig. S3, D and E); similarly, the wake-promoting effect of optogenetic stimulation was significantly reduced by the preceding sensory stimulation (fig. S3, F and G). This is consistent with our finding that repeated sensory and optogenetic stimulation both lead to functional fatigue of LC-NE neurons, which can reduce the wake-promoting effect of succeeding stimulation.

### α_2_A receptor knockdown reduces LC-NE functional fatigue and extends wakefulness

**Fig. 4.**
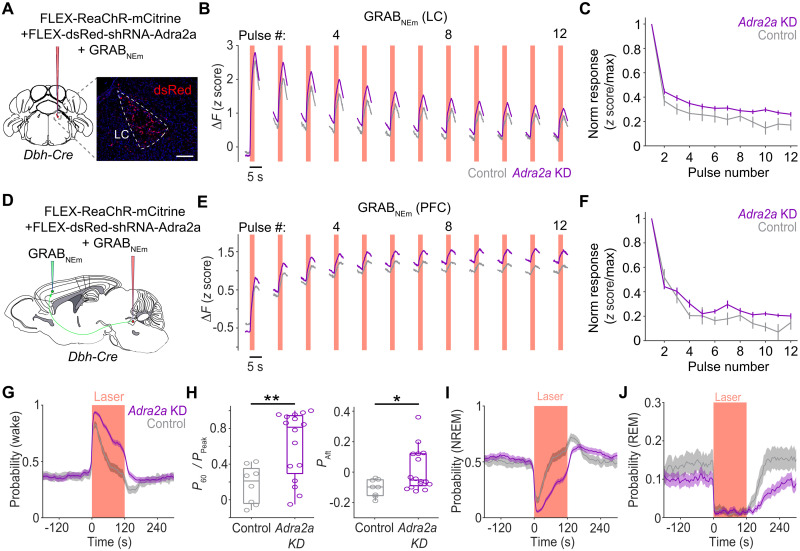
Effects of *Adra2a* knockdown on evoked NE release and sleep-wake behavior. (**A**) Diagram for AAV injection of the Cre-inducible *Adra2a* knockdown virus with AAVs expressing ReaChR-mCitrine and GRAB_NEm_ into the LC of *Dbh-Cre* mice; fluorescence image shows dsRed-shRNA-*Adra2a* expression in LC-NE neurons (fluorescence in situ hybridization). (**B**) Evoked NE release (LC) by 2-s pulses averaged across all mice for control (gray, from Fig. 2G; *n* = 7 mice) and *Adra2a* knockdown (KD) mice (purple, *n* = 10 mice). (**C**) Summary of GRAB_NE_ (LC) response size (normalized by the maximum in each episode, mean ± SEM); normalized NE responses were significantly different between groups (*P* < 0.05, Scheirer-Ray-Hare test). (**D**) Diagram for Cre-inducible *Adra2a* knockdown with coexpression of ReaChR-mCitrine and GRAB_NEm_ in LC and GRAB_NEm_ in mPFC. (**E**) Evoked NE release (mPFC) for control (gray, from Fig. 2I; *n* = 6 mice) and *Adra2a* knockdown mice (purple, *n* = 4 mice). (**F**) Normalized GRAB_NE_ (mPFC) response size; significant difference between groups (*P* < 0.05, Scheirer-Ray-Hare test). (**G**) Wake probability relative to bilateral laser stimulation (2-s pulse beginning every 10 ± 5 s) in control (gray, from fig. S1C; *n* = 8 mice) and *Adra2a* knockdown mice (purple, *n* = 16 mice). (**H**) Persistence of laser-evoked wake effect (*P*_60_/*P*_Peak_ ratio; see Fig. 1E, left) and recovery sleep (*P*_Aft_; see Fig. 1E, right) in control and *Adra2a* knockdown mice. Each point represents a mouse average. *P < 0.05, **P < 0.01 (Wilcoxon rank-sum test). (**I** and **J**) NREM and REM probabilities relative to bilateral laser stimulation (*n* = 8 control mice, 16 *Adra2a* knockdown mice).

What cellular processes mediate the rapid decline of LC neuron calcium responses and NE release? A possible mechanism is autoinhibition induced by released NE, which binds to α_2_A adrenergic receptors and causes hyperpolarization of LC neurons following their activation (*28*, *29*). To test this possibility, we knocked down the expression of α_2_A receptor (*Adra2a*) specifically in LC-NE neurons using short hairpin RNA (shRNA). The dsRed-shRNA-*Adra2a* construct, shown to be effective for *Adra2a* knockdown in vitro and in vivo (*29*), was inserted into a Cre-inducible AAV (AAV5-FLEX-dsRed-shRNA-Adra2a; fig. S4A) and injected into the LC of *Dbh-Cre* mice together with AAVs expressing ReaChR and GRAB_NEm_ (Fig. 4A). Costaining for Th and dsRed using fluorescence in situ hybridization showed that 47 ± 3% of LC-NE neurons (mean ± SEM, *n* = 3 mice) were transfected with dsRed-shRNA-Adra2a. The expression of α2A receptor was significantly reduced in transfected neurons (fig. S4, B and C). Compared to control mice (Fig. 2, G to J), the decline in NE release evoked by successive optogenetic stimulation was attenuated in *Adra2a* knockdown mice in both the LC and mPFC (Fig. 4, B to F). Furthermore, *Adra2a* knockdown significantly prolonged the waking effect of LC-NE activation (Fig. 4, G and H) and delayed the increases in NREM (Fig. 4I) and REM sleep (Fig. 4J). The overall REM sleep probability was also reduced during the recovery phase in *Adra2a* knockdown mice.

## DISCUSSION

Here, we have shown that activation of LC-NE neurons with either optogenetic or sensory stimulation caused rapid declines of their calcium responses and evoked NE release (Figs. 2 and 3), which may contribute to the increase in sleep propensity (Fig. 1) and sleep depth (fig. S1H). In principle, increased sleep after stimulating wake-promoting neurons could be a general homeostatic response to the induced wakefulness rather than caused specifically by the stimulated neurons. In our study, however, a rapid increase in NREM sleep was induced by stimulating LC-NE but not vM-Glu, POA-GABA, or BF-ChAT neurons [studied previously (*21*–*23*)], although their wake-promoting effects were similar during the early part of the stimulation period (Fig. 1, D and E). This suggests that a strong increase in wakefulness was not sufficient to cause the subsequent increase in sleep; instead, the effect depends on the stimulated cell type. In addition to vM-Glu, POA-GABA, and BF-ACh neurons, activating dopaminergic neurons in the dorsal raphe also caused wakefulness that outlasted a 2-min activation period (*30*). In future studies, it would be interesting to examine the time course of brain state modulation by other cell types as well.

Previous studies of LC-NE neurons often focused on their wake-promoting effect (*15*–*17*). In one study, 3-Hz optogenetic activation for 1 hour caused an overall increase in wakefulness (*15*). Here, we show that activation of LC-NE neurons using a variety of protocols [including 3-Hz stimulation used in (*15*); fig. S1E] causes only a transient wakefulness [consistent with their reported role in microarousals (*17*)], which is followed by a marked increase in sleep pressure. This highlights a previously understudied role of LC-NE neurons in sleep homeostasis. The discrepancy between the previous and current studies may be partly due to the use of *Th-IRES-Cre* mice in the earlier study (*15*), which labels a larger population of neurons than the *Dbh-Cre* line, including some wake-promoting neurons that are adjacent to but outside of the LC (*17*, *26*, *31*).

We found that the highly transient wake-promoting effect of LC-NE neurons, distinct from several other arousal-promoting neuronal populations that induce sustained wakefulness (Fig. 1, D and E), is mediated in part by autoinhibition through α_2_A receptors (Fig. 4). Given the time course of G_i_-coupled α_2_A receptor signaling, this mechanism is well suited for regulating sleep homeostasis on a timescale of minutes, similar to the timescale of the natural sleep-wake cycle in mice (*32*). Such a negative feedback mechanism could also contribute to the infraslow oscillation of LC-NE activity (*17*, *33*). In addition to direct effects on LC neurons through α_2_A receptors, released NE can also affect many other neurons, astrocytes, and microglia (*34*), which, for example, may lead to increased adenosine that could inhibit LC-NE neurons (*35*) and increase sleep pressure (*6*–*8*). It would be interesting to explore how manipulations of these pathways may affect activity-induced decline of LC-NE activity and homeostatic sleep regulation.

In general, sleep pressure could be driven by an increase in sleep-promoting factors (e.g., adenosine) and/or weakening of wake-promoting mechanisms, such as functional fatigue of LC-NE neurons. LC-NE neurons are known to be particularly vulnerable to neurodegeneration, exhibiting earlier and greater cell loss than many other neurons in both Alzheimer’s and Parkinson’s diseases (*36*–*38*). An intriguing possibility is that their susceptibility to functional fatigue is mechanistically linked to their vulnerability to degeneration. The rapid decline of LC-NE functional efficacy and the resulting increase in sleep pressure could offer a protective mechanism against the harmful effects of excessive LC-NE activation. On the other hand, it could also reflect sensitivity of these neurons to activity-induced metabolic stress (e.g., oxidative stress caused by reactive oxygen species) (*39*) that may contribute to both functional fatigue and neurodegeneration (*40*, *41*). Thus, the dynamic regulation of LC-NE activity may represent a key link in the bidirectional relationship between sleep impairment and neurodegeneration (*42*–*44*).

## MATERIALS AND METHODS

### Animals

All procedures were performed in accordance with the protocol approved by the Animal Care and Use Committee at the University of California, Berkeley (AUP 2016-06-8860). Adult (6 to 12 weeks old) male and female *Dbh-Cre* mice [B6.FVB(Cg)-Tg(Dbh-cre)KH212Gsat/Mmucd, MMRRC: 036778-UCD] were used for all experiments. Mice were kept on a 12:12 light:dark cycle. After virus injections and surgical implantation of EEG/EMG electrodes and optical fibers, mice were individually housed to prevent damage to the implant before experiments. Experiments were conducted 2 to 4 weeks after surgery.

### Surgeries

Anesthesia was induced with 5% isoflurane and maintained with 1.5% isoflurane on a stereotax. Buprenorphine (0.1 mg/kg, subcutaneously) and meloxicam (10 mg/kg, subcutaneously) were injected before surgery. Lidocaine (0.5%, 0.1 ml, subcutaneously) was injected near the target incision site. Body temperature was kept stable throughout using a heating pad and a feedback thermistor. After sterilizing the skin with ethanol and betadine, a circular patch of skin and connective tissue was cut away to expose the skull. Surgeries typically consisted of virus injection followed by EEG/EMG implantation and fiber implantation.

For virus injections, a craniotomy was drilled above the target site (see below for details of virus injections). Ten minutes after virus injection, the injection pipette was slowly removed from the injection site. For recording the EEG and EMG, stainless-steel wires were soldered to a 20-pin header (Minitek 127T series). EEG screw electrodes were implanted in both sides of the frontal lobe [(±1.5 mm mediolateral (ML), −1.5 mm anteroposterior (AP)] to leave space for the optic fibers implanted into the LC. EMG stainless-steel wire electrodes (0.003-inch diameter) were inserted into both sides of the trapezius muscle. Reference screws were inserted into each side of the cerebellum. The EEG/EMG implant was secured by dental cement before fiber implantation. For fiber photometry and optogenetic stimulation, a fiber implant (1.25-mm ferrule, 200-μm core) was held by a stereotactic fiber holder (XCL, Thor Labs). Either single (Neurophotometrics) or dual fibers (TFC_200/245-0.37_6mm_TS1.8_FLT, Doric Lenses Inc.) were implanted 100 μm above the virus injection site. Fibers were secured with cyanoacrylate glue and dental cement before withdrawing the stereotactic fiber holder.

### Virus injections

Injections were performed using Nanoject II (Drummond Scientific) with glass pipettes. The injection settings were set to a 50-nl injection volume at a rate of 23 nl/s with a 30-s interval between injections. AAV2-hSyn-FLEX-ReaChR-mCitrine [0.3 × 10^13^ to 3.0 × 10^13^ genome copies/milliliter (gc/ml, 50955 from Addgene)] was injected bilaterally or unilaterally into the LC of Dbh-Cre mice [−5.5 AP, ±0.9 ML, 250 nl at −3.7 dorsoventral (DV) and 250 nl at −3.5 DV]. For the control in fig. S1J, AAV5-synP-DIO-GFP (0.4 × 10^13^ gc/ml, 100043 from Addgene) was injected bilaterally into the LC of Dbh-Cre mice. For fiber photometry experiments, the ReaChR virus was coinjected unilaterally into the LC with AAV9-hSyn-FLEX-jGCaMP8m (3 × 10^13^ gc/ml, 162378-AAV9 from Addgene) or with AAV9-hSyn-GRABNEm (2 × 10^13^ to 9 × 10^13^ gc/ml, YL003008-AV9 from WZ Biosciences). For photometry in the mPFC, AAV9-hSyn-GRABNEm was injected unilaterally (250 nl, +2.1 AP, +0.3 ML, −1.6 DV).

### EEG/EMG recording

EEG/EMG electrodes were connected to a custom recording cable (P1 Technologies) that interfaced with data acquisition hardware (S-Box-16, PZ-5, RZ5 from Tucker-Davis Technologies). Mice were habituated overnight, and recording sessions started at 6:30 a.m. and lasted 6 hours. Each mouse was typically tested for three sessions. The first session was preceded by a single night of habituation, but later sessions were run after the mouse has been in the same environment for multiple days. In a small number of mice, the EEG/EMG became unstable or detached, resulting in only one or two recorded sessions. Data were acquired using Synapse software (version 95-44132P, Tucker-Davis Technologies), with a digital band-pass filter of 1 to 750 Hz and a sampling rate of 1500 Hz. Classification of sleep-wake behavioral state in each 5-s epoch was performed according to published methods (*34*). Briefly, wakefulness was characterized by high muscle tone (root mean square of EMG). NREM sleep was characterized by low muscle tone and high EEG δ power (sum of power at 1 to 5 Hz). REM sleep was characterized by low δ power, low muscle tone, and high θ power (sum of power at 6 to 9 Hz)/δ power ratio. The classification was semiautomatic with visual inspection using a graphical user interface in MATLAB (MathWorks).

### Optogenetic stimulation

To study the transient wakefulness induced by activation of LC-NE neurons, we applied laser stimulation (532 or 589 nm, 10 mW at fiber tip). Recording sessions consisted of 24 stimulation episodes (first stimulation at 8:00 a.m.—1 hour after chamber light onset, interstimulation-episode-onset-interval of 10 min). Each stimulation episode lasted 2 min and consisted of either intermittent pulse protocols [2-s stimulation of either constant light pulses (Figs. 1 and 2 and fig. S1, A and C), 10-ms pulses at 5 Hz (fig. S1D), 10-ms pulses at 3 Hz (fig. S1E), or 10-ms pulses at 1 Hz (fig. S1F) every 10 ± 5 s] or a 10-Hz protocol [10-ms pulse at 10 Hz (Fig. 1 and fig. S1B)] controlled by transistor-transistor logic (TTL) pulses from an RZ5 BioAmp Processor (Tucker-Davis Technologies). Three 6-hour sessions were typically recorded from each mouse.

### Fiber photometry

Fiber photometry was performed using a complementary metal-oxide semiconductor (CMOS) camera–based system (FP3001, Neurophotometrics and Bonsai data acquisition software, bonsai-rx.org), with 470- and 405-nm light-emitting diodes (LEDs). The sampling rate for each channel was 10 frames/s. The system was turned on for 70 min to equilibrate the camera and bleach autofluorescence before collecting experimental data. For analysis of jGCaMP8m and GRAB_NEm_ signals, we fit the 470- and 405-nm channels to a biexponential decay function to approximate the slow photobleaching time course (*45*). The biexponential fit was subtracted from each channel, and the 405-nm channel was fit to the 470-nm channel using a least squares method (*45*). To correct artifacts unrelated to changes in calcium or norepinephrine (NE), we subtracted the fitted 405-nm (isosbestic) signal from the 470-nm (ligand-dependent) signal (*45*). The final corrected 470-nm signal was then *z*-scored. For the example trace in Fig. 2F, to better visualize the slow NE responses, the photometry signal was low pass–filtered at 0.2 Hz, which eliminated fast noise without altering the response shape.

### Mechanical stimulation

An Internet-of-Things relay device (Digital Loggers) was used to control motion (~60 rpm) of an orbital shaker (MT-201-BD, Labnique) with a TTL pulse from an RZ5 BioAmp Processor (Tucker-Davis Technologies). The home cage of the mouse was placed on a sheet of aluminum foil grounded to the recording equipment through the PZ-5 grounding port (Tucker-Davis Technologies). Mechanical stimulation often introduced sharp artifacts in the photometry data that were clearly distinguishable from the relatively slow calcium and NE fluctuations. To remove these artifacts, we calculated the derivative of the fluorescence signals and removed data points that rose above a threshold value.

### Cre-dependent knockdown of *Adra2a*

We generated a Cre-dependent shRNA virus (AAV5-EF1α-FLEX-dsRed-shRNA-Adra2a) targeting mouse *Adra2a* by synthesizing a linear DNA insert (gBlock from Integrated DNA Technologies) consisting of dsRed and an shRNA sequence, which has been validated for *Adra2a* knockdown in vitro and in vivo (*29*). The hairpin structure hindered DNA synthesis, so the plasmid was constructed by inserting the dsRed-shRNA-Adra2a with two gBlocks by inserting half of the insert in each gBlock. Site-directed mutagenesis (New England Biolabs) was used to remove the cloning scar. The linear DNA insert was ligated in reverse complement orientation into a Cre-inducible AAV plasmid with an EF-1α promoter and a 3′ Woodchuck Hepatitis Virus Posttranscriptional Regulatory Element (DNA sequence available at https://osf.io/q38ca). The plasmid was sequenced using long-read sequencing (Nanopore) and packaged into AAV5 (BRAIN Initiative Viral Vector Core). The shRNA-Adra2a virus (6.70 × 10^12^ gc/mL) was mixed with ReaChR and GRAB_NEm_ at a ratio of 1:1:1 or 2:1:1 in the injection mixture. Virus expression in LC-NE neurons was checked using in situ hybridization to colabel dsRed and TH (481361-C2 and 317621-C3, Advanced Cell Diagnostics).

### Histology

To evaluate the location and strength of virus expression as well as the fiber position, mice were anesthetized with isoflurane before transcardial perfusion with 15 ml of Dulbecco’s phosphate-buffered saline (DPBS) (14200-075, Gibco) and 30 ml of 4% paraformaldehyde (15714, Electron Microscopy Sciences) in DPBS. For fiber localization, the bottom of the skull was removed to expose the ventral surface of the brain and the brain was postfixed in 4% paraformaldehyde DPBS for one to two nights before separating the brain from the top of the skull. The fixed brain was dehydrated in 15 ml of 30% sucrose DPBS for one to two nights, allowing time for it to sink to the bottom of a conical tube. The brain was then embedded in tissue freezing medium (General Data Company Inc.) and frozen in a −80°C freezer for at least 1 hour. Brains were cryosectioned at 40 μm using a cryostat (Leica). ReaChR-mCitrine fluorescence was visible without immunostaining. Nonspecific binding was blocked with 10% normal donkey serum in PBS-T (pH 7.2, 0.1% Tween 20). To identify the LC region, cryosections were immunostained with a rabbit TH antibody (ab112, Abcam, 1:300 in blocking solution). To evaluate jGCaMP8m and GRABNEm expression, cryosections were immunostained with a chicken anti-GFP antibody (GFP-1020, Aves Labs, 1:100). Brain sections were stained for one night at 4°C. Donkey antirabbit antibodies conjugated to red or far-red Alexa fluorophores (A10042, Invitrogen; ab150067, Abcam) were used to visualize TH staining. A donkey antichicken antibody conjugated to Alexa Fluor 488 (A78948, Thermo Fisher Scientific) was used to visualize GFP staining. Secondary antibodies were incubated for 2 hours at room temperature. Slides were washed with PBS-T, mounted with Aqua-Poly/Mount (18606, Polysciences), and imaged on a fluorescence microscope (Keyence).

### Quantification of *Adra2a* expression in control in *Adra2a* knockdown mice

We performed fluorescence in situ hybridization (323100 RNAscope Multiplex Fluorescent Reagent Kit v2, ACD Bio) to label mRNA molecules for *Th* (317621-C3, ACD Bio), *Adra2a* (425341, ACD Bio), and *dsRed* (481361-C2, ACD Bio) in sections of the LC. To quantify the efficiency of Adra2a knockdown, defined as the percentage reduction in the puncta density across all infected LC-NE neurons versus control mice not injected with the shRNA-Adra2a virus, we counted *Adra2a* puncta in *Th*-positive LC neurons from three control mice (44 LC-NE neurons total) and three *Adra2a* knockdown mice (42 LC-NE neurons total). Transfected neurons in *Adra2a* knockdown mice were identified by *dsRed* expression. Fluorescent images were taken with a laser-scanning confocal microscope using a 63× oil-immersion objective (Zeiss). Individual *Adra2a* puncta were counted using deconvolution and an automated cell-segmentation function “cellpose” (*46*, *47*) implemented in MATLAB, with some manual correction. Punctal density was quantified as the number of puncta in each LC-NE neuron divided by the area of the *Th*-positive cell body.

### Quantification and normalization of calcium and NE responses

For each optogenetic or mechanical stimulation pulse, the response was quantified by calculating the mean signal from 0 to 1 s after stimulation onset minus the mean signal from −1.5 to 0 s before stimulation onset. This short response window was chosen because the mechanical stimulation experiments involved two revolutions of the orbital shaker within the 2-s pulse (60 rpm), which often produced two peaks in the calcium and NE responses, whereas the optogenetic stimulation was a single square pulse, which typically evoked a single peak (within the 0- to 1-s window for calcium responses). The response for each pulse in the episode was averaged across all stimulation episodes in a recording session. For normalization, the average response for each pulse was divided by the maximum average response from the episode.

### Statistics

We performed the Shapiro-Wilk test for normality and determined that all datasets could not be treated as normally distributed, and thus, we used nonparametric statistics for all figures (table S1). Sleep-wake probability plots and transition probabilities were analyzed as described previously (*21*). Briefly, the 95% confidence intervals (CIs) for brain state probabilities and statistical tests were calculated from bootstrapped data as follows: For *n* mice, we calculated the CI by randomly resampling the data (with replacement) for 10,000 iterations. Then, we recalculated the mean probabilities for each sleep-wake state across the mouse averages for the *n* mice. The lower and upper CIs were then extracted from the distribution of the bootstrapped data. To test whether a given transition probability was significantly larger or smaller from the baseline at stimulation onset or at recovery onset, we performed bootstrap significance tests comparing the target bin and the baseline average. All other statistical tests are shown in table S1. No statistical method was used to predetermine sample size, but our sample sizes are similar to those reported in previous publications (*21*–*23*).
