# Supplementary material for: Activation of locus coeruleus noradrenergic neurons rapidly drives homeostatic sleep pressure
Source: Sci Adv. 2025 Jan 17;11(3):eadq0651. doi: 10.1126/sciadv.adq0651 (PMC11740930; doi:10.1126/sciadv.adq0651)
Supplement: Supplementary file 1 — Figs. S1 to S4 Table S1 [file sciadv.adq0651_sm.pdf]

Science Advances
AAAS

Supplementary Materials for

Activation of locus coeruleus noradrenergic neurons rapidly drives homeostatic sleep pressure

Daniel Silverman *et al.*

Corresponding author: Yang Dan, ydan@berkeley.edu

Sci. Adv. **11**, eadq0651 (2025)
DOI: 10.1126/sciadv.adq0651

This PDF file includes:

Figs. S1 to S4
Table S1

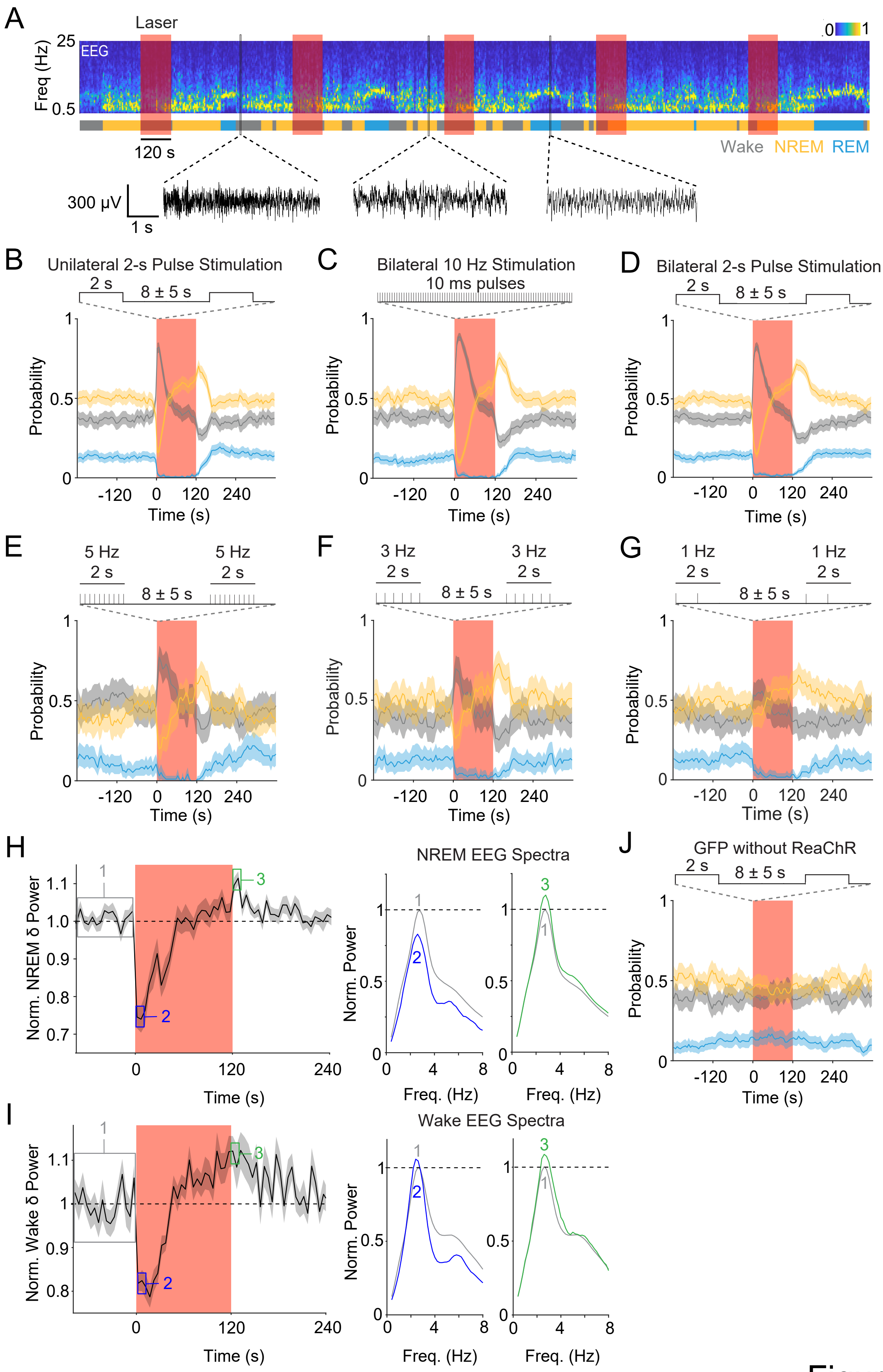

A
Laser
EEG
Freq (Hz)
25
0.5
0
1
120 s
Wake NREM REM
300 µV
1 s
B
Unilateral 2-s Pulse Stimulation
2 s
8 ± 5 s
C
Bilateral 10 Hz Stimulation
10 ms pulses
D
Bilateral 2-s Pulse Stimulation
Probability
Time (s)
-120
0
120
240
E
5 Hz
F
3 Hz
G
1 Hz
H
Norm. NREM δ Power
NREM EEG Spectra
Norm. Power
Freq. (Hz)
I
Norm. Wake δ Power
Wake EEG Spectra
J
GFP without ReaChR

Figure S1

Fig. S1. Effects of different patterns of LC-NE neuron optogenetic activation on brain states and NREM δ power. **(A)** Representative EEG traces during wake, NREM, and REM sleep for recording session shown in Fig. 1B. **(B)** Probability of wake, NREM and REM states before, during, and after unilateral LC stimulation (2-s pulses every 10 ± 5 s, n = 8 mice). **(C,D)** similar to **(B)**, with bilateral stimulation: 10 ms pulses at 10 Hz, n = 8 mice **(C)** or 2-s pulses every 10 ± 5 s, n = 8 mice **(D). (E-G)** similar to **(B-D)**, with 2-s stimulation periods every 10 ± 5 s consisting of 10 ms pulses at 5 Hz, **(E)**, 3 Hz **(F)** or 1 Hz **(G)** (n = 3 mice tested with each protocol). **(H) Left,** EEG δ power during NREM sleep before, during, and after LC stimulation, averaged across all sessions (n = 44 sessions, 8 mice). **Right,** NREM EEG power spectra within three time windows: before stimulation (1, gray box), 5 sec after stimulation onset (2, blue), and 5 sec after stimulation (3, green), normalized by the peak in window 1. The average NREM δ power during the 120 s after stimulation termination was significantly elevated above the baseline during the 120 s before stimulation onset (One-sided Wilcoxon rank-sum test, $p < 0.01$, n = 8 mice, **Table S1**). **(I)** similar to **(H)**, but for EEG δ power during wake. The average wake δ power during the 120 s after stimulation termination was significantly elevated above the baseline during the 120 s before stimulation onset (One-sided Wilcoxon rank-sum test, p < 0.01, **Table S1**). **(J)** Bilateral stimulation in control mice expressing GFP without ReaChR, with stimulation protocol similar to **(D)** (n = 3 mice)**.**

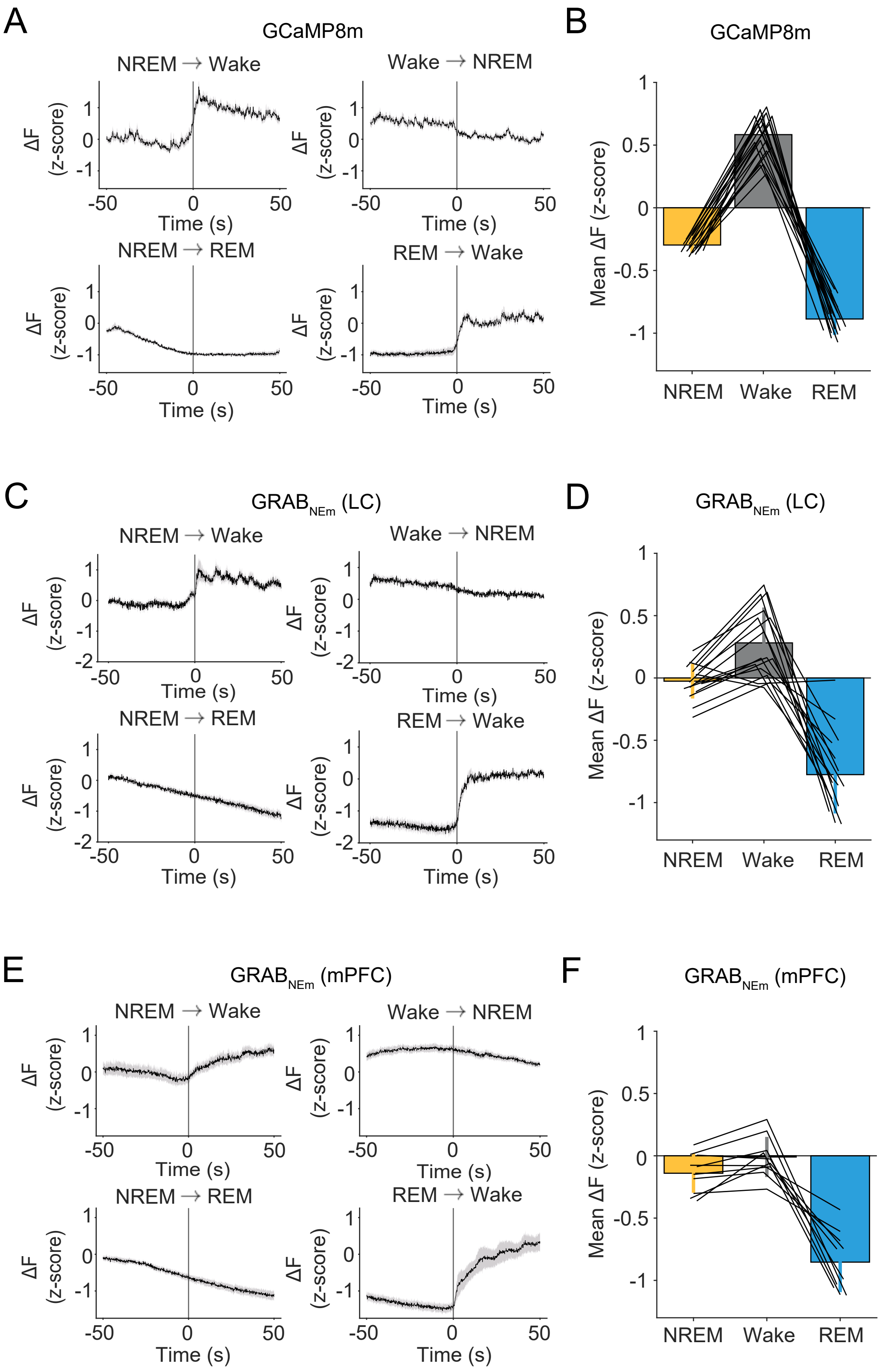

A
GCaMP8m
NREM → Wake
Wake → NREM
NREM → REM
REM → Wake
ΔF (z-score)
Time (s)
B
GCaMP8m
Mean ΔF (z-score)
NREM
Wake
REM
C
GRAB$_{NEm}$ (LC)
D
GRAB$_{NEm}$ (LC)
E
GRAB$_{NEm}$ (mPFC)
F
GRAB$_{NEm}$ (mPFC)

Figure S2

Fig. S2. LC-NE neuron activity at sleep-wake state transitions

(A) Calcium signal in LC-NE neurons before and after each state transition. **(B)** Mean calcium signal during each state (mean ± SEM), each line represents one session (n = 19 sessions, 6 mice). **(C-F)** Similar to **(A,B)** but for NE levels in the LC (**C,D**; n = 16 sessions, 7 mice) and mPFC (**E,F**; n = 10 sessions, 6 mice).

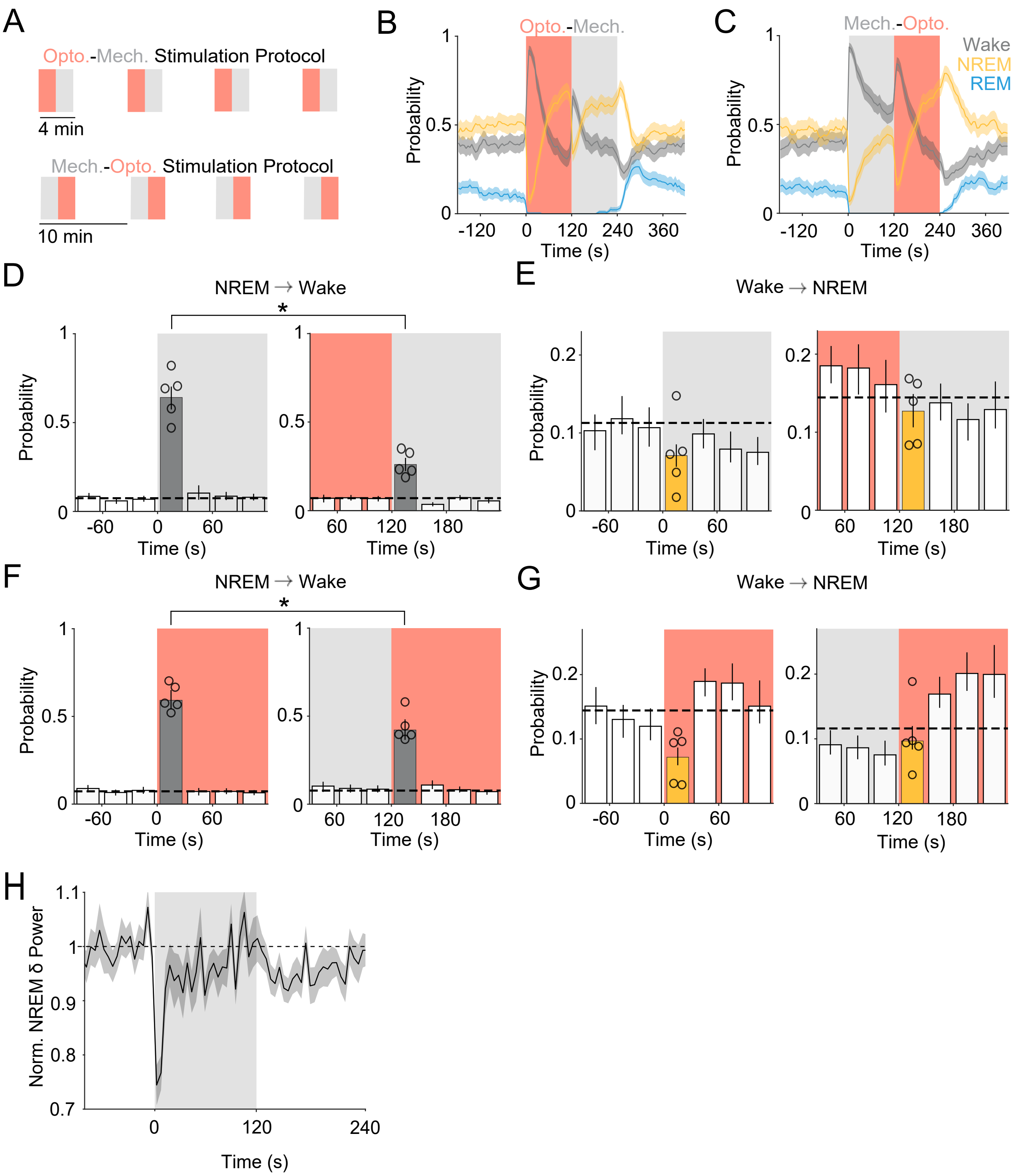
A
Opto.-Mech. Stimulation Protocol
4 min
Mech.-Opto. Stimulation Protocol
10 min
B
Opto.-Mech.
Probability
Time (s)
C
Mech.-Opto.
Wake
NREM
REM
Probability
Time (s)
D
NREM → Wake
Probability
Time (s)
E
Wake → NREM
Probability
Time (s)
F
NREM → Wake
Probability
Time (s)
G
Wake → NREM
Probability
Time (s)
H
Norm. NREM δ Power
Time (s)

Figure S3

Fig. S3. Effects of consecutive optogenetic and mechanical stimulation.

(A) Schematic for consecutive optogenetic and mechanical stimulation episodes. **(B,C)** Probability of each state before, during, and after optogenetics-mechanical stimulation **(B)** and mechanical-optogenetic stimulation **(C)**. **(D,E)** NREM→Wake **(D)** and Wake→NREM **(E)** transition probabilities at mechanical stimulation onset with mechanical-optogenetic (left) or optogenetic-mechanical protocol (right). The increase in NREM→Wake probability evoked by mechanical stimulation is significantly suppressed by the preceding optogenetic stimulation. **(F,G)** Similar to **(D,E)** showing that NREM→Wake probability evoked by optogenetic stimulation is significantly suppressed by the preceding mechanical stimulation, /, $p < 0.05$. (Wilcoxon signed-rank test, n = 5 mice, 3 sessions with each protocol). **(H)** EEG δ power during NREM sleep before, during, and after mechanical stimulation, averaged across all sessions (n = 45 sessions, 15 mice).

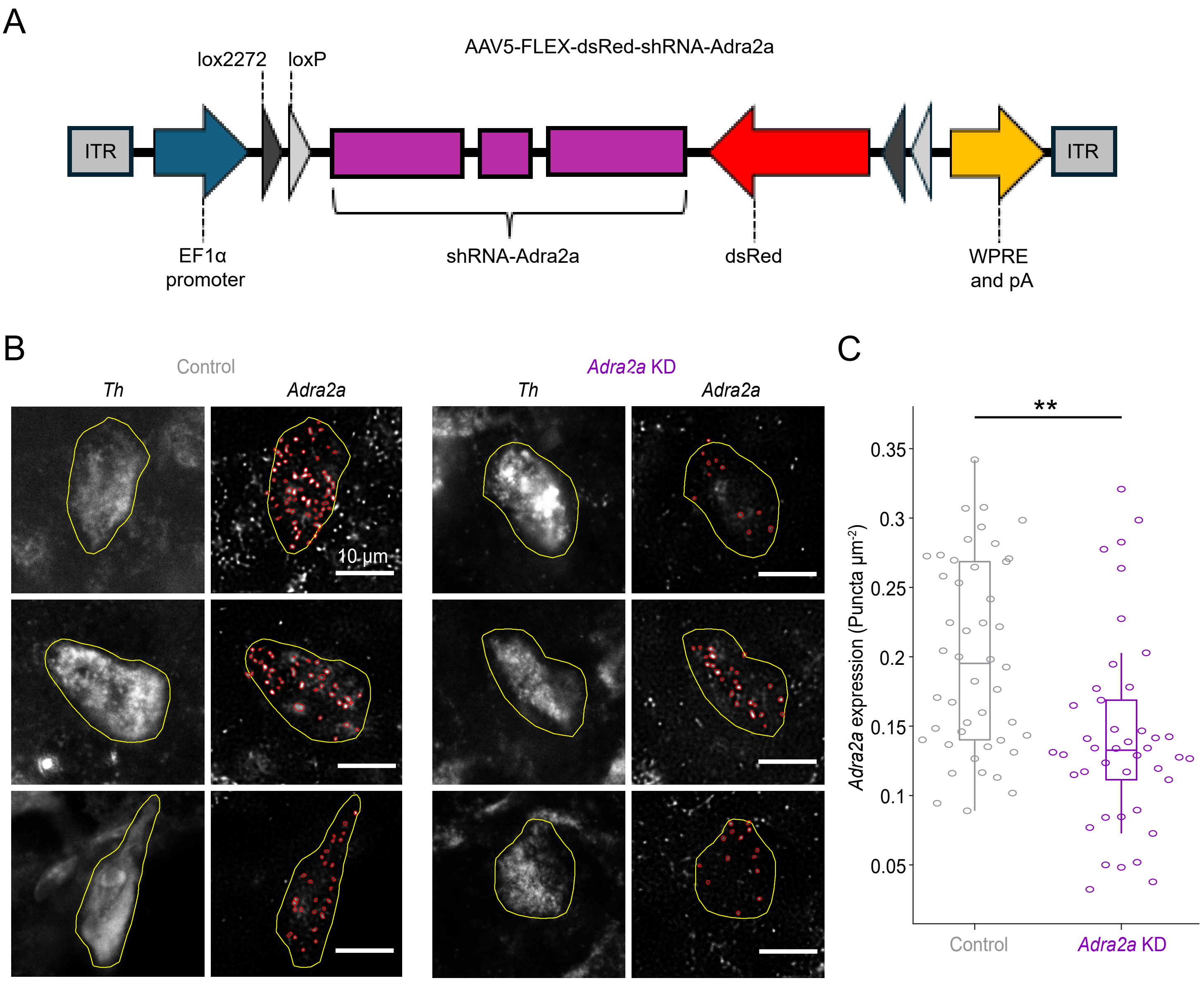
A
AAV5-FLEX-dsRed-shRNA-Adra2a
lox2272
loxP
ITR
ITR
EF1α
promoter
shRNA-Adra2a
dsRed
WPRE
and pA
B
Control
Th
Adra2a
Adra2a KD
Th
Adra2a
10 µm
C
**
Adra2a expression (Puncta µm-2)
0.35
0.3
0.25
0.2
0.15
0.1
0.05
Control
Adra2a KD

Figure S4

Fig. S4. Quantification of *Adra2a* knockdown efficiency with fluorescence *in situ* hybridization

(A) Schematic of AAV-FLEX-dsRed-shRNA-Adra2a DNA construct. **(B)** Representative LC-NE neurons from control (Left) and *Adra2a* KD mice (Right). Each row is from a different mouse. The cell bodies of LC-NE neurons were identified by *Th* FISH labeling (outlined in yellow), and *Adra2a* mRNA puncta (outlined in red) were counted. **(C)** Adra2a expression in control and Adra2a KD mice. Each point represents the number of *Adra2a* mRNA puncta divided by the area of each LC-NE neuron, **, $p < 0.01$.

Figure	Sample Size	Statistical Test	Results
Figure 1F	8 mice , 2 stimulation protocols each (4 males, 4 females)	Bootstrap	After stimulation onset<br>95% confidence Interval =<br>0.26-0.30, $p < 0.0001$ (N→W);<br>0.03-0.04, $p < 0.0001$ (W→N)
Figure 1G	8 mice, 2 stimulation protocols each (4 males, 4 females)	Bootstrap	After stimulation termination<br>95% confidence Interval =<br>0.03-0.04, $p < 0.0001$ (N→W);<br>0.23-0.28, $p < 0.0001$ (W→N)
Figure 3C (Left)	15 mice (11 males, 4 females)	Bootstrap	After stimulation onset (Left)<br>95% confidence Interval =<br>0.53-0.61, $p < 0.0001$ (N→W);<br>0.08-0.10, $p < 0.0001$ (W→N)
Figure 3C (Right)	15 mice (11 males, 4 females)	Bootstrap	After stimulation termination (Right)<br>95% confidence Interval =<br>0.03-0.04, $p < 0.0001$ (N→W);<br>0.22-0.27, $p < 0.0001$ (W→N)
Figure 3J (Top)	6 mice, GCaMP8m (3 males, 3 females)<br>7 mice, $GRAB_{NE}$ (LC) (4 males, 3 females)<br>6 mice, $GRAB_{NE}$ (PFC) (3 males, 3 females)	One-sided Wilcoxon rank-sum test	W = 63, $p = 0.0006$, GCaMP8m vs. $GRAB_{NE}$ (LC),<br>W = 57, $p = 0.001$, GCaMP8m vs. $GRAB_{NE}$ (mPFC)
Figure 3J (Bottom)	5 mice, GCaMP8m (4 males, 1 female)<br>5 mice, $GRAB_{NE}$ (LC) (4 males, 1 female)<br>6 mice, $GRAB_{NE}$ (PFC) (3 males, 3 females)	One-sided Wilcoxon rank-sum test	W = 37, $p = 0.028$, GCaMP8m vs. $GRAB_{NE}$ (LC),<br>W = 42, $p = 0.015$, GCaMP8m vs. $GRAB_{NE}$ (mPFC)
Figure 4C	7 control mice, (4 males, 3 females)<br>10 *Adra2a* KD mice (7 males, 3 females)	Scheirer-Ray-Hare test	H = 6.41, $p = 0.011$<br>(Group comparison:*Adra2A* KD vs. Control)<br>H = 30.3, $p = 0.0008$<br>(Pulse comparison)<br>H = 1.55, $p = 0.999$<br>(Group x Pulse Interaction)
Figure 4F	6 control mice, (3 males, 3 females)<br>4 *Adra2a* KD mice (4 males)	Scheirer-Ray-Hare test	H = 4.53, $p = 0.033$<br>(Group comparison: *Adra2A* KD vs. Control)<br>H = 41.36, $p < 0.0001$<br>(Pulse comparison)<br>H = 2.59, $p = 0.99$<br>(Group x Pulse Interaction)

Figure 4H	8 control mice,<br>(4 males, 4 females)<br>16 *Adra2a* KD mice<br>(13 males, 3 females)	One-sided Wilcoxon rank-sum test	W = 60, p = 0.0078 for P_{60} / P_{Peak}<br>W = 69, p = 0.031 for P_{Aft}<br>(*Adra2A* KD vs. Control)
Figure S1H, Left	8 mice<br>(4 males, 4 females)	One-sided Wilcoxon rank-sum test	Average normalized NREM δ Power in 120 s after stimulation termination compared to average normalized NREM δ Power in 120 s before stimulation onset<br>W = 469, p = 0.007<br>Average normalized wake δ Power in 120 s after stimulation termination compared to average normalized wake δ Power in 120 s before stimulation onset<br>W = 1698, p = 0.002
Figure S3 D,E	5 mice<br>(1 male, 4 females)	One-sided Wilcoxon signed-rank test	W = 15, p = 0.03 (NREM-to-wake)<br>W = 4, p = 0.22 (wake-to-NREM)
Figure S3 F,G	5 mice<br>(1 male, 4 females)	One-sided Wilcoxon signed-rank test	W = 0; p = 0.03 (NREM-to-wake)<br>W = 14, p = 0.06 (wake-to-NREM)
Figure S4 B	44 control LC-NE neurons<br>(2 males, 1 female)<br>42 *Adra2a KD* LC-NE neurons<br>(3 males)	One-sided Wilcoxon signed-rank test	W = 2326; p = 0.0002 (*Adra2A* KD vs. Control)

Table S1. Test statistics and *p*-values for all figures.
